# A state-wide population-based evaluation of cervical cancers arising during opportunistic screening in the United States

**DOI:** 10.1016/j.ygyno.2020.08.033

**Published:** 2020-11

**Authors:** Rebecca Landy, Christopher Mathews, Michael Robertson, Charles L. Wiggins, Yolanda J. McDonald, Daniel W. Goldberg, Isabel C. Scarinci, Jack Cuzick, Peter D. Sasieni, Cosette M. Wheeler

**Affiliations:** a Wolfson Institute of Preventive Medicine, Queen Mary University of London, London, United Kingdom; b Division of Cancer Epidemiology and Genetics, National Cancer Institute, National Institutes of Health, Department of Health and Human Services, Bethesda, MD, USA; c School of Cancer & Pharmaceutical Sciences, King's College London, London SE1 9RT, United Kingdom; d University of New Mexico Comprehensive Cancer Center, The Center for HPV Prevention, University of New Mexico Health Sciences Center, Albuquerque, NM, USA; e University of New Mexico Comprehensive Cancer Center, Department of Internal Medicine, University of New Mexico Health Sciences Center, Albuquerque, NM, USA; f Department of Human and Organizational Development, Vanderbilt University, Nashville, TN, USA; g Department of Geography, Texas A&M University, College Station, TX, USA; h Division of Preventive Medicine, University of Alabama at Birmingham, AL, USA; i Departments of Pathology and Obstetrics & Gynecology, University of New Mexico Health Sciences Center, Albuquerque, NM, USA

**Keywords:** Cervical cancer, Cytology, Human papillomavirus (HPV), Screening, New Mexico, Case series

## Abstract

**Objective:**

Despite widespread cervical screening, an estimated 13,800 women will be diagnosed with cervical cancer in the United States in 2020. To inform improvements, the screening histories of women diagnosed with cervical cancer in New Mexico were assessed.

**Methods:**

Data were collected on all cervical screening, diagnostic tests and treatment procedures for all women diagnosed with cervical cancer aged 25-64 yrs. in New Mexico from 2006 to 2016. Women were categorized by their screening attendance in the 5–40 months (screening interval) and 1–4 months (peri-diagnostic interval) prior to cancer diagnosis.

**Results:**

Of the 504 women diagnosed between May 2009–December 2016, 64% were not screened or had only inadequate screening tests in the 5–40 months prior to diagnosis, and 90 of 182 screened women (49%) had only negative screens in this period. Only 32% (N = 162) of cervical cancers were screen-detected. Women with adenocarcinomas were more likely to have had a recent negative screen (41/57 = 722%) than women with squamous cancers (50/112 = 45%). Both older women (aged 45–64 years) and women with more advanced cancers were less likely to have been screened, and if screened, were more likely to have a false-negative outcome. Only 9% of cancers were diagnosed in women who did not attend biopsy or treatment after positive tests requiring clinical management. Screening currently prevents 35% of cancers, whereas full screening coverage could prevent 61% of cervical cancers.

**Conclusion:**

Improved screening coverage has the largest potential for reducing cervical cancer incidence, though there is also a role for improved recall procedures and screening sensitivity.

## Highlights

• 64% of women diagnosed with cervical cancer aged 25-64y had not been screened in the 3-years prior to diagnosis.
• Screening currently prevents 35% of cancers; full screening coverage could prevent 61% of cancers.
• One-third of cervical cancers were screen-detected (i.e. diagnosed within 4 months of a positive screen).
• 72% of the adenocarcinomas had a negative screen in a 3-yr period prior to diagnosis.
• 9% of cancers were associated with a failure to receive recommended diagnostic biopsy or treatment (failsafe failure).

## 1 Introduction

Cervical screening, which is widely available in the United States (U.S.), is an effective, although imperfect, method of preventing cervical cancer. It works by detecting precancerous lesions which can be removed, preventing progression to cancer. Previously, screening was carried out using cytology. Recently, HPV (human papillomavirus) testing has been introduced, which is more sensitive for detecting precancerous lesions [1]. When HPV testing and cytology are performed together, this is known as co-testing. The consensus guidelines for cervical screening in the U.S. issued in 2012 recommended screening begin at age 21 yrs., with 3-yearly cytology for women aged 21-29 yrs., and 3-yearly cytology or 5-yearly co-testing for women 30-64 yrs. [2,3].

An estimated 13,800 women will be diagnosed with cervical cancer in the United States in 2020, and 4290 women will die from it [4]. It is crucial to understand why so many women still develop cervical cancer despite screening: had they attended screening? If so, was the cancer missed; was the woman lost to follow-up? Or did the cancer occur despite previous treatment of a pre-cancer? Cancers diagnosed at stage IA can be considered a partial success of screening; although not prevented, they were diagnosed at a very early stage, where the treatment is often almost the same as for cervical intraepithelial neoplasia grade 3 (CIN3), and the 5-year relative survival rate is 98.1% [5]. Previous research has shown that screening is less effective at preventing adenocarcinomas than squamous cancers, though screening does detect some adenocarcinomas at an earlier stage [6].

We examined the screening histories of women diagnosed with cervical cancer in New Mexico from 2009 to 2016, with the aim of identifying where the largest gains can be made in preventing cervical cancer.

## 2 Methods

Women diagnosed with cervical cancer in New Mexico from 1 January 2006–31 December 2016 were identified through linkage of the New Mexico Tumor Registry (NMTR) and the New Mexico HPV Pap Registry (NMHPVPR). Since 2006, under New Mexico's reporting requirements for Notifiable Diseases and Conditions, screening (cytology and HPV) tests, as well as diagnostic and treatment biopsies must be reported to the New Mexico HPV Pap Registry (NMHPVPR). We used combined data from NMTR and NMHPVPR to document each woman's cancer diagnosis, screening history and treatment events. Items included outcomes (HPV positive/negative, cytology results, diagnostic/excisional biopsy with CIN grade reported, and hysterectomy), as well as histology and stage at cancer diagnosis (using the derived AJCC6 classification system [7]). A measure of urbanisation, the 2010 (revised July 3rd 2019) rural-urban commuting area (RUCA) code (most urban: RUCA 1–3, most rural: RUCA 8–10) [8], was derived based on the woman's address of residence at diagnosis.

Over the study period the vast majority of screening was by cytology alone, although co-testing was increasingly used from 2013 to 2016; because the recommendation in late 2012 was for 5-year co-testing, only positive co-tests within 40 months of diagnosis are considered and the screening history of some cancers with an antecedent negative co-test may not have been included in our analysis. As only (laboratory) test results must be reported to NMHPVPR under state regulation, confirmation of colposcopy was only possible when a biopsy was performed and therefore we could not differentiate between failure to attend colposcopy and attendance where no biopsy was taken.

To better understand the separate roles of screening (per se) and colposcopy and treatment in the resultant cancers, we considered events in the 5–40 month window prior to cancer diagnosis separately from what happened in the peri-diagnostic period (1–4 months prior to diagnosis). Essentially there are three possibilities in the screening window: no screening, only negative screening, or positive screening. If the woman was referred to colposcopy as a result of screening, she might: not attend colposcopy, have cancer diagnosed via colposcopy, or have cancer diagnosed despite having attended colposcopy. Among women not referred to colposcopy in the screening window, we can further classify them by whether in the peri-diagnostic interval they had: no screening; negative screening; or screening resulting in referral to colposcopy.

To ensure a full 40 months of screening history, we restrict our analyses to women diagnosed between 1 May 2009 and 31 December 2016 (i.e. 40 months from 1 January 2006).

### 2.1 Classifying screening histories

Our classification of screening histories is based on the results of tests both 5–40 months prior to diagnosis, as well as in the peri-diagnostic period (Flowchart 1). Women with no screening test (or only inadequate samples) in the 40 months prior to diagnosis were classified as not screened (coverage failure). These cases, considered inadequately screened, were further sub-categorized into two categories: coverage failure-screen detected (inadequately screened) for those with a positive test only in the peri-diagnostic period, or coverage failure-inadequately screened for those with no or only negative tests in the peri-diagnostic period. We consider a screening test to be negative if the cytology result was negative (provided there was not also a concurrent positive HPV co-test), or if the cytology result was atypical squamous cells of undetermined significance (ASC-US) with a negative HPV test due to triage of ASC-US or a co-test.

Women who attended screening were categorized depending on whether their results indicated referral to colposcopy. Colposcopy was recommended during the study period based on a single positive test (ASC-US cytology with a positive HPV co-test, or LSIL+ (low-grade squamous intraepithelial lesion or worse) cytology regardless of any HPV result), or a repeat abnormality on a repeat test at a short interval. Lesser non-negative results (ASC-US cytology with no HPV co-test, or a positive HPV result without an abnormal cytology result (ASC-US or worse) [9]) elicit a repeat screening test at a short interval. If a woman had a biopsy recorded within the 6 months following a screening result which would indicate a repeat test at a short interval, this woman was considered to have been referred to colposcopy.

Women with either only negative screening tests or tests indicating a repeat test at a short interval in the 5–40 months prior to diagnosis were considered to be screened – no colposcopy referral. If there were no screening test results available prior to the first test result indicating a repeat test at a short interval, we assumed it was a first test indicating a repeat test at a short interval if they did not have a biopsy in the following 6 months. The screened – no colposcopy referral women were further sub-categorized into two categories: screening test failure for those who had either no testing or only negative testing in the peri-diagnostic period and screen detected (adequately screened) for those who had a positive test in the peri-diagnostic period.

Women with a positive screening test result which should have referred them directly to colposcopy in the 5–40 months prior to diagnosis were considered to be screened – positive (post-test failure). Those women for whom there is no record of attending colposcopy in the following 6 months (i.e. there is no biopsy record) were considered failsafe failures (no biopsy) (Flowchart 2). These could be either women who were never seen at colposcopy or who had a colposcopy, but no biopsy was taken. The remaining women attended colposcopy within 6 months of their referring screen. If they were then diagnosed with cancer within 6 months of their first or second biopsy, provided it was within 6 months of a referring screen or biopsy, these women were considered to have screen detected (adequately screened) cancers. The final group are termed post-referral failures. These include: women for whom the most severe biopsy within 6 months of the referring screen was negative or CIN1 (colposcopy failure); women whose most severe biopsy within 6 months of the referring screen was CIN2/3 but who were not treated within 6 months (failsafe failures (no treatment)); and those women who developed cancer despite treatment (treatment failure).

Analyses were restricted to women aged 25-64 yrs. at diagnosis, to ensure they were eligible for screening for the previous 40 months. Two-sided Z-tests were carried out to compare proportions. The proportion of cancers that could have been prevented if all women had attended screening was estimated, using previously published odds ratios for stage I and stage II+ cancer among women in New Mexico who attended screening in a 3-year period compared to women who did not (0.62 and 0.22 respectively) [10]. The number of cancers that would have occurred in the absence of screening was estimated using previously published methodology (supplementary material) [11]. Additionally, we estimated the number of cancers that would have been associated with each screening history category had there been 100% screening coverage (supplementary material).

Our study was reviewed and determined exempt by the University of New Mexico Human Research Review Committee. Reporting under state regulations (New Mexico Administrative Code) specified by the list of Notifiable Diseases and Conditions.

## 3 Results

There were 614 women diagnosed with cervical cancer in New Mexico between May 2009 and December 2016. Fifteen (2.4%) were diagnosed in women <25 yrs., and 95 (15%) were diagnosed in women 65 yrs. and older, the age when women with an adequate negative screening history are recommended to exit screening [2]. This left 504 women diagnosed aged 25-64 yrs.; the basic clinical and sociodemographics are shown in Table 1.

**Table 1:** Cervical screening in the 5–40 months prior to diagnosis for women diagnosed with cervical cancer aged 25–64 years in New Mexico May 2009–December 2016.

	A. Not screened	B. Screened - negative	C. Screened - positive	Total (%)	% not screened^b^	Of screened, % negative^c^
Total	322	100	82	100%	64%	55%
Age (years)						
25–34	43	26	32	20%	43%	45%
35–44	100	24	30	31%	65%	44%
45–54	48	11	8	13%	72%	58%
55–64	131	39	12	36%	72%	76%
FIGO stage						
IA	50	25	34	22%	46%	42%
I NOS	17	1	7	5%	68%	13%
I B	50	35	16	20%	50%	69%
II	34	10	4	10%	71%	71%
III	90	16	8	23%	79%	67%
IV	49	8	5	12%	79%	62%
Unknown	32	5	8	9%	71%	38%
II+	173	34	17	44%	77%	67%
Morphology						
Squamous	243	50	62	70%	68%	45%
Adenocarcinoma	45	41	16	20%	44%	72%
Other	34	9	4	9%	72%	69%
Race/Ethnicity						
Non-Hispanic White	138	50	40	45%	61%	56%
White Hispanic	141	36	33	42%	67%	52%
Native American	29	10	5	9%	66%	67%
Other/unknown	14	4	4	4%	64%	50%
Health insurance						
Private	99	48	25	34%	58%	66%
Medicaid	101	17	30	29%	68%	36%
Medicare and other Government	25	14	6	9%	56%	70%
Not insured	41	4	6	10%	80%	40%
Unknown	56	17	15	17%	64%	53%
RUCA^a^						
Most urban (RUCA 1–3)	199	69	57	64%	61%	55%
RUCA 4–7	89	24	20	26%	67%	55%
Most rural (RUCA 8–10)	34	7	5	9%	74%	58%

a Rural-urban commuting area, based on address at diagnosis.

b A/(A + B + C).

c B/(B + C).

The age distribution of cervical cancers in New Mexico is very similar to the age distribution for cervical cancers diagnosed in the US overall [12]. Similarly, the histological distribution was similar to the US overall, with 70% squamous, and 20% adenocarcinoma. Of those with known stage, 51% were stage I (24% IA, 5% I-NOS, 22% IB) and 49% were stage II+ (10% stage II, 25% stage III, 14% stage IV). This distribution is very similar to the US overall.

Forty-five percent of women diagnosed were non-Hispanic Whites, 42% Hispanic Whites, 9% Native Americans, and 4% other/unknown. This is a similar to the population living in New Mexico according to 2010 census data [13]. Forty-one percent of women with known insurance status had private insurance, 36% had Medicaid, and 12% were uninsured. Nine percent of the women lived in the most rural areas (areas with a RUCA code 8–10).

**Fig. 1:**
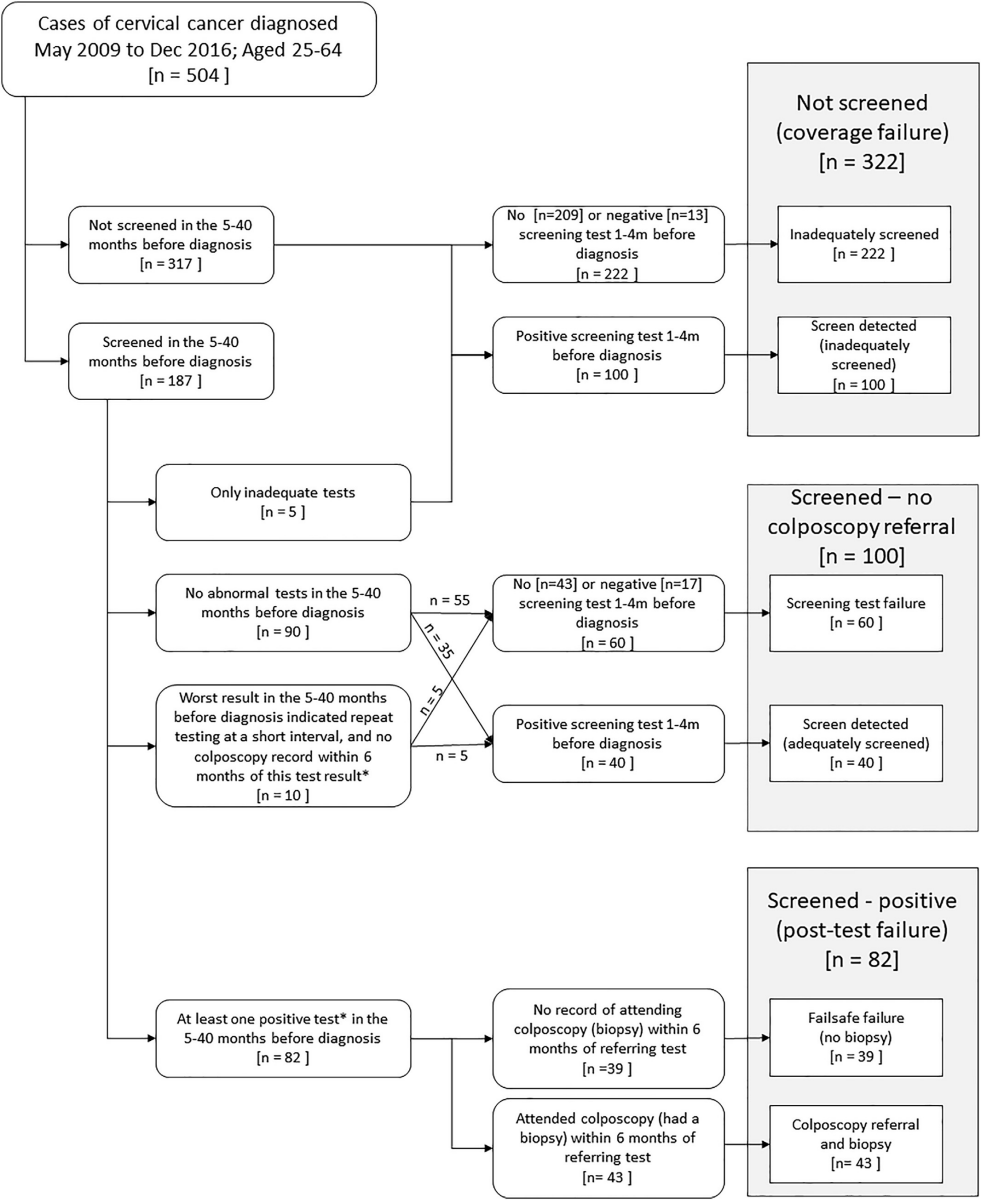
Flowchart of the cervical cancer screening algorithm. *A positive test is defined to be a test which should have referred a woman directly to colposcopy. An HPV positive with no abnormal cytology or ASCUS/LSIL cytology without a positive HPV test indicate repeat testing at a short interval. If a woman has a biopsy record after a test result indicating a repeat test at a short interval, they are treated as if they had a positive test, as it may have been the second such result, which would indicate a colposcopy referral.

Among the 322 coverage failures, 317 had no screening tests in the 5–40 months prior to diagnosis (63% of all cases), and 5 women (1% of all cases) had only inadequate tests in this period (Fig. 1). Of these 322 women, 100 (31%) had a positive screening test in the peri-diagnostic period and were classified as screen detected (inadequately screened) (Table 2, Supplementary Table 1).

**Table 2:** Screening history classification for women diagnosed with cervical cancer aged 25–64 years in New Mexico, May 2009–December 2016.

	A. Inadequately screened^a^		B. Screen detected (inadequately screened)^a^		C. Screen detected (adequately screened)^a^		D. Screening test failure^a^		E. Failsafe failure (no biopsy)^a^		F. Post colposcopy failure^a^		% screen detected^d^	of adequately screened, % screen detected^e^
	N	Row %	N	Row %	N	Row %	N	Row %	N	Row %	N	Row %		
Total	222	44%	100	20%	62	12%	60	12%	39	8%	21	4%	32%	34%
Age (years)														
25–34	24	24%	19	19%	17	17%	15	15%	17	17%	9	9%	36%	29%
35–44	66	43%	34	22%	22	14%	12	8%	14	9%	6	4%	36%	41%
45–54	38	57%	10	15%	8	12%	5	7%	3	4%	3	4%	27%	42%
55–64	94	52%	37	20%	15	8%	28	15%	5	3%	3	2%	29%	29%
FIGO stage														
IA	16	15%	34	31%	26	24%	13	12%	11	10%	9	8%	55%	44%
IB^b^	44	35%	23	18%	19	15%	19	15%	13	10%	8	6%	33%	32%
II+	136	61%	37	17%	13	6%	24	11%	11	5%	3	1%	22%	25%
Unknown	26	58%	6	13%	4	9%	4	9%	4	9%	1	2%	22%	31%
Morphology														
Squamous	159	45%	84	24%	41	12%	29	8%	29	8%	13	4%	35%	37%
Adenocarcinoma	33	32%	12	12%	18	18%	25	25%	8	8%	6	6%	29%	32%
Other	30	64%	4	9%	3	6%	6	13%	2	4%	2	4%	15%	23%
Race/Ethnicity														
Non-Hispanic White	90	39%	48	21%	27	12%	31	14%	21	9%	11	5%	33%	30%
White Hispanic	99	47%	42	20%	29	14%	19	9%	13	6%	8	4%	34%	42%
Native American	25	57%	4	9%	3	7%	8	18%	2	5%	2	5%	16%	20%
Other/unknown	8	36%	6	27%	3	14%	2	9%	3	14%	0	0%	41%	38%
Health insurance														
Private	75	44%	24	14%	28	16%	29	17%	9	5%	7	4%	30%	38%
Medicaid	63	43%	38	26%	13	9%	9	6%	18	12%	7	5%	34%	28%
Medicare and other Government	18	40%	7	16%	4	9%	11	24%	3	7%	2	4%	24%	20%
Not insured	26	51%	15	29%	3	6%	3	6%	4	8%	0	0%	35%	30%
Unknown	40	45%	16	18%	14	16%	8	9%	5	6%	5	6%	34%	44%
RUCA^c^														
Most urban (RUCA 1–3)	136	42%	63	19%	47	14%	38	12%	26	8%	15	5%	34%	37%
RUCA 4–7	63	47%	26	20%	13	10%	16	12%	11	8%	4	3%	29%	30%
Most rural (RUCA 8–10)	23	50%	11	24%	2	4%	6	13%	2	4%	2	4%	28%	17%

a For definitions of screening classifications, see Fig. 1, Fig. 2.

b FIGO stage IB includes stage I not otherwise specified.

c Rural-urban commuting area, based on address at diagnosis.

d B + C.

e C/(C + D + E + F).

Of the 182 women with an adequate screening test in the 5–40 months prior to diagnosis, 90 (49%) had only negative tests, and 10 (5%) had a screening test result indicating a repeat test at a short interval, giving a total of 100 women (55%) who were screened – no colposcopy referral. Of these 100 women, 40% also had a positive test in the peri-diagnostic period (screen detected (adequately screened)), whereas 60 women had either no test or only negative tests (test failure).

Of the 82 women (16%) who had a screening test result which should have referred them to colposcopy (Fig. 2), 58 (71%) had a biopsy within 12 months, including 43 women (52%) who had a biopsy within 6 months of the referring screen. Half of these 43 women (N = 22, 51%) had cancer diagnosed within 6 months of the biopsy (screen detected (adequately screened)), 28% (N = 12) had a negative/CIN 1 biopsy (colposcopy failure), and 21% (N = 9) had CIN2/3 diagnosed on biopsy. Of the 9 women with CIN2/3, 4 were not treated within 6 months of CIN2/3 diagnosis (failsafe failure (no treatment)); the other 5 had an excisional treatment within 6 months, but nevertheless developed cancer (treatment failure). These 5 women had positive margins on pathology review. The remaining 24 of the 82 women who should have been referred to colposcopy either did not attend colposcopy or attended but did not have a biopsy taken.

**Fig. 2:**
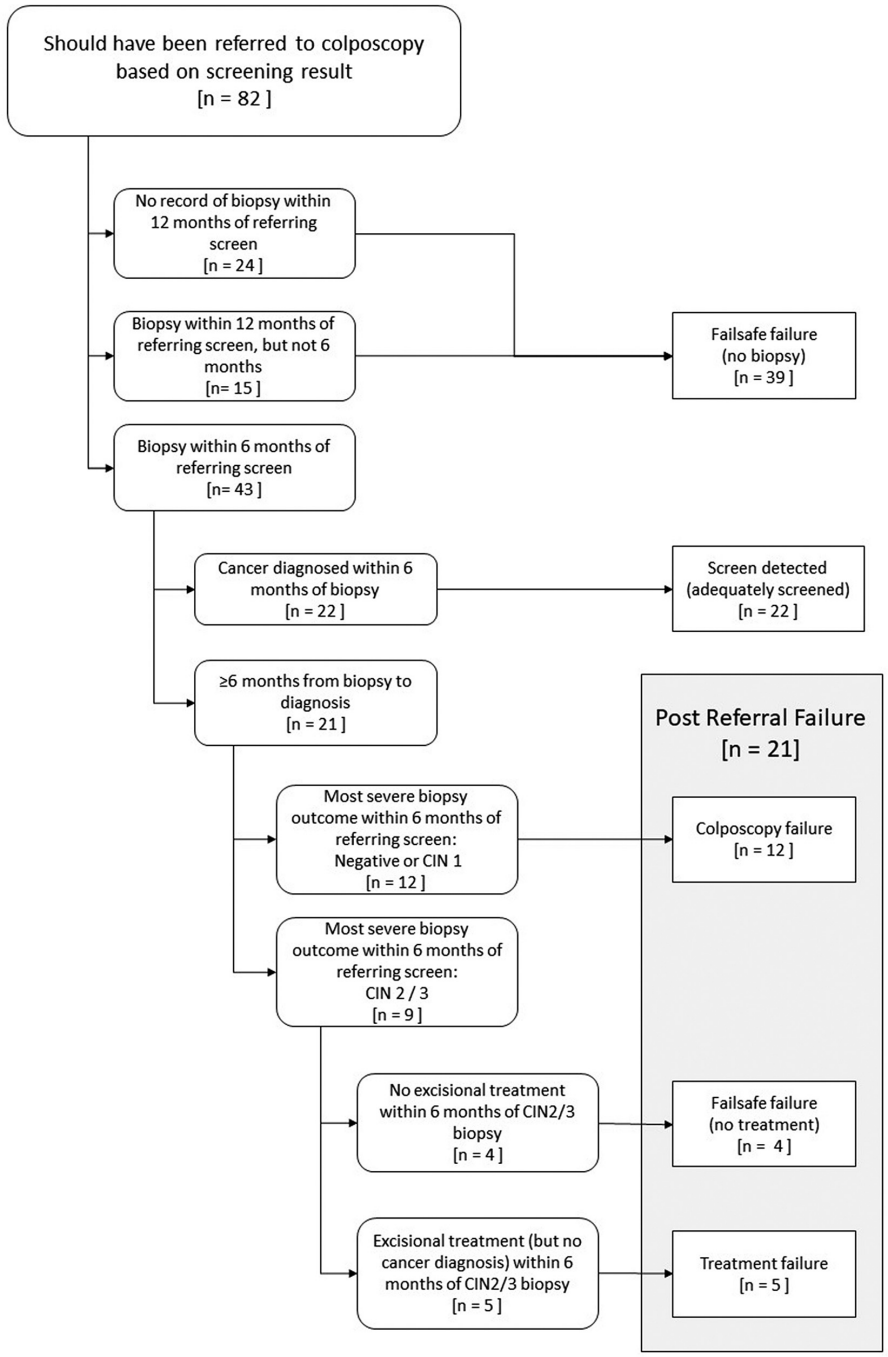
Flowchart of the referral algorithm following a positive cervical screening test.

Overall, 322 (64%) women were not screened, 100 (20%) were screen negative, and 82 (16%) had a screening result which should have referred them directly to colposcopy (i.e. were screen positive) (Table 1). Sixty-two women (12% of all 504 cancers) were screened during the three-year interval and had a positive test in the peri-diagnostic period: they are classified as screen-detected (adequately screened). Thirty-nine women (8%) had failsafe failures since they did not have a biopsy following an abnormal screening test (Table 2), and 21 (4%) had a post-referral failure, including 12 (2%) who had a colposcopy failure, 5 (1%) had a treatment failure and 4 (1%) who did not have a treatment after a biopsy indicating CIN2/3 (Fig. 2).

Overall, 162 (32%) women had a positive test in the peri-diagnostic period (screen detected), but only 62 of these women were adequately screened prior to this. Of the 182 adequately screened women with cervical cancer, one third (N = 62) were screen-detected, and a third (N = 60) developed cancer without an intervening positive test (screening test failure). The remaining third (N = 60) developed cancer despite a previous positive screen (39 failsafe failures and 21 post-colposcopy failures).

**Fig. 3:**
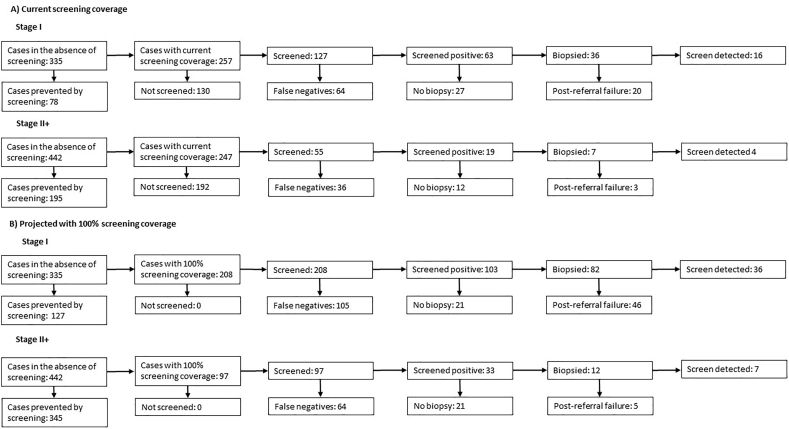
Projected number of cancers associated with each screening history (A) under current screening coverage and (B) with 100% screening coverage.

In the absence of screening, we estimate that there would have been 777 cancers diagnosed in the same period in New Mexico. Screening is therefore currently preventing 35% of cervical cancers, and increasing screening coverage to 100% has the potential to prevent 61% of all cancers (Table 3). Fig. 3 shows the number of women who would have a false negative screen, who would not have a biopsy, and who would have a post-referral failure under current screening coverage (Fig. 3A), and the projected values with 100% screening coverage (Fig. 3B).

**Table 3:** Estimated number of cancers in the absence of screening and in the presence of 100% screening coverage.

	N cancers		Overall		
	Stage I	Stage II+	N cancers	Ratio relative to current screening	Ratio relative to no screening
Cancers in absence of screening	334.9	441.7	776.6	1.54	1
Cancers with current screening	257.0	247.0	504.0	1	0.65
Cancers with 100% screening coverage	207.6	97.2	304.8	0.60	0.39

### 3.1 Age

The lowest proportion of unscreened women were 25–34 yrs. (25–34 yrs.:43%, 35–44 yrs.:65%, 45–54 yrs.:72%, 55–64 yrs.:72%, p-value for trend <0.001, Table 1). There was also a significant increasing trend with age in the proportions of screened women whose screen was negative (p-value < 0.001, Table 1). A slightly higher proportion of younger women's cancers were screen-detected (25–44 yrs.:36% vs 45–64 yrs.:28%), though when considering only adequately screened women who were diagnosed with cervical cancer, this proportion was higher for women aged 35–54 yrs. (41%) than women aged 25–34 yrs. or 55–64 yrs. (both 29%) (Table 2).

### 3.2 Stage

As stage at diagnosis increased, so did the proportion of women who were unscreened (Table 1), from 46% of women with stage IA cancer to 79% with stage IV (p-value for trend <0.001). Of the screened women, women with more advanced cancers were more likely to have a negative screen (stage IA:42%, stage II+:67%, p-value for trend <0.001). The proportion of screen-detected cancers was much higher for stage IA cancers than stage II+ cancers (55% vs 22%), and this difference remained when accounting for the proportion who were screened (p-value for trend 0.008) (Table 2).

### 3.3 Morphology

The proportion unscreened was higher among women with squamous cancers compared to adenocarcinomas (68% vs 44%, p < 0.001). Of the screened women, women with adenocarcinomas were more likely to have had a negative screening test (72% vs 45%, p < 0.001).

### 3.4 Race/ethnicity

Among women with cervical cancer, the proportion unscreened was similar across race/ethnicity. Screened Native American women were slightly more likely to have a negative result (67% vs 56% of non-Hispanic White women), though numbers were very low (10/15 women). Similarly, screened Native American women were slightly less likely to have a screen detected cancer (20%), though again numbers were low (3/15).

### 3.5 Health insurance status

Women without health insurance were least likely to have been screened (80% unscreened), followed by women with Medicaid (68% unscreened). However if women with these health insurance statuses were screened, they were least likely to have a false negative screen (40% and 36% respectively, compared to 66% of women with private insurance and 70% of women with Medicare or other Government insurance). The overall proportion of women whose cancers were screen detected was similar across health insurance statuses (24%–35%), though accounting for the proportion of women who were screened, women with private insurance were slightly more likely to have their cancers screen detected (38% vs 20%–30%).

### 3.6 Rurality

A non-significantly decreasing proportion of women had attended screening with increasing rurality (p-value for trend 0.053), from 39% of the most urban women to 26% of the most rural. However the same proportion of screened women had a positive screening result. Overall, the same proportion of the cancers were screen-detected regardless of rurality, but once accounting for the proportion of women who were adequately screened, the proportion who had a screen-detected cancer decreased with increasing rurality, from 37% of the most urban women to 17% of the most rural women (p-value for trend 0.099).

### 3.7 HPV vs cytology testing

Of the 182 women with an adequate screening test in the 5–40 months prior to diagnosis, 71 had an adequate HPV test, and 181 women had an adequate cytology test. 64% of women with an adequate cytology test had a false-negative test, compared to 23% with an adequate HPV test. Of the women with squamous cancers, 53% (N = 59/111) of women with an adequate cytology test had a false-negative test, compared to 22% (N = 10/45) with an adequate HPV test. For women with adenocarcinomas, 82% (N = 47/57) of women with an adequate cytology test had a false-negative test, compared to 24% (N = 5/21) with an adequate HPV test.

### 3.8 Co-testing

54 women had a co-test as their first screening test in the 5–40 months prior to diagnosis. 12 women (22%) had negative HPV tests, of whom 7 (58%) had abnormal cytology, and 14 women (26%) had negative cytology tests, of whom 9 (64%) had a positive HPV test. Five women were negative on both tests.

## 4 Discussion

We have classified the screening histories of all 504 women diagnosed with cervical cancer aged 25-64 yrs. in the state of New Mexico between May 2009 and December 2016. Two-thirds of the women diagnosed did not have an adequate screen in a 3-year period prior to diagnosis. Based on previously published odds ratios for cervical cancer comparing screened to unscreened women in New Mexico, we estimate that screening currently prevents 35% of cancers. If screening coverage was increased to 100%, we estimate that screening has the potential to prevent 61% of cancers. This demonstrates that work needs to be done at all stages of the screening process to prevent cervical cancers, not just increasing screening coverage - further reductions could be achieved through increasing test sensitivity or reducing failsafe failures. Nearly a quarter (24%) of women with cancer were diagnosed despite what appeared to be appropriate screening and management. Half of these cancers (12% of all cancers) were screen-detected in adequately screened women (and over 70% of these were stage I), and half (12% altogether) were diagnosed after adequate screening without a colposcopy referral (screening test failure). The final 12% of cancers were not screen-detected and developed despite a screening referral to colposcopy: 8% did not have a biopsy, and the remaining 4% had either a colposcopy or treatment failure, or failed to be treated despite a biopsy result of CIN2/3.

It is important to know why women develop cervical cancer despite the wide availability of screening, to optimise the use of resources in reducing the incidence. Given the majority of women in our study had not attended screening in the previous 3 years, increasing cervical screening coverage can play a major role in preventing cervical cancer among women beyond the age for which HPV vaccination is recommended. The recommended screening interval for co-testing is 5 yrs.; 53% of cases in New Mexico with 5 years of screening history data were not screened in the 5 yrs. prior to diagnosis. The introduction of self-sampling may help increase screening coverage, though it is important to ensure that failsafe failure rates do not increase due to a lack of safety nets in place for women who test HPV positive on a self-sample but do not attend appropriate follow-up. In New Mexico, a failsafe failure was involved in 9% of cancers, mainly relating to colposcopy attendance following an abnormal screening test (8%), with 4 women (1%) failing to attend a repeat colposcopy for excisional treatment following a positive biopsy. Safety netting is likely to remain a challenge for the U.S. given the vast majority of health care delivery settings lack call/recall systems and established procedures to reduce failsafe failures.

Our results agree with other studies which have found a higher proportion of women with adenocarcinomas have been screened than women with squamous cancers [6,14]. The proportion of women with adenocarcinoma with an adequate cytology test who had a negative cytology test within 5–40 months prior to diagnosis was 82%, compared to 24% of women with an adequate HPV test who had a negative HPV test. This implies that the observed change in the last few years in New Mexico from cytology alone to either HPV testing alone or co-testing could greatly reduce the proportion of women with a cervical adenocarcinoma (or precursors to adenocarcinoma) who have a false negative test.

Overall, 100 women diagnosed with cervical cancer had a false negative screening test. The sensitivity of HPV testing to pre-cancer is 97% among women who test cytology positive [15]. If the sensitivity is slightly lower (95%) among women who test negative on cytology, this would imply that 95 of the 100 women who tested cytology negative would test HPV positive. An upper limit of the proportion of cancers in women aged 25–64 which could be prevented by implementing primary HPV testing is therefore 19% (95/504) of the cancers in the absence of vaccination which aren't already prevented by screening, which is 12% (95/777) of the estimated total number of cancers in the absence of vaccination or screening. This is an upper limit of the number of cancers that would be prevented if HPV primary testing completely replaced cytology, as some of the women who tested cytology-positive and whose cancers were prevented would have a false-negative HPV, and their cancer may not be prevented, and others would already have had occult cancer at the time of their negative cytology test. This estimate also assumes appropriate management of all women following their positive screening test. However as well as decreasing screening test failures, a switch to HPV testing will increase the workload for colposcopists, as women can be referred to colposcopy with no cytologic abnormalities, but either a single HPV 16/18 positive HPV test or two consecutive positive HPV tests [16].

Results were very similar across race/ethnicity, though the number of Native American women diagnosed with cervical cancer is low, limiting our ability to identify any differences in the screening histories of Native American women who developed cervical cancer. Only one-fifth of women without health insurance coverage who were diagnosed with cancer had attended screening in a 3-year period prior to diagnosis. Increasing usage of the National Breast and Cervical Cancer Early Detection Program (NBCCEDP), which provides breast and cervical screening, as well as diagnostic and treatment services, to low-income and uninsured/underinsured women could reduce the proportion of uninsured women who are unscreened.

There is a long history of interest in understanding the screening histories of women who developed cervical cancer. Numerous studies from around the world have found non-attendance at screening to be a key reason for the development of cancer despite the availability of cervical screening [[17], [18], [19], [20], [21], [22], [23]]. Castle et al. [24] recently described the screening histories of 623 women diagnosed with cervical cancer aged ≥30 yrs. in 2003–15 in one integrated health care system (Kaiser Permanente Northern California; KPNC) in the U.S. Since this study was restricted to women with a co-test result preceding the diagnosis, we cannot compare the proportion who were unscreened. However, among screened women who had an incident cancer, some results are broadly comparable; the KPNC study found a lower proportion of screened women had a false negative screen in the 1-4 yrs. prior to diagnosis than in New Mexico (27% (N = 70/263) vs 49% (N = 90/182)). Overall three times as many women in KPNC had a false-negative diagnosis at colposcopy in the 1-5 yrs. prior to diagnosis (21% (N = 56/263) vs 7% (N = 12/182)). A very similar proportion of women in both studies did not have a biopsy when referred to colposcopy (17% in KPNC, 16% in our study).

Since we only have records of biopsies rather than colposcopy attendance, some women may have attended colposcopy without having a biopsy. However 2006 colposcopy guidelines recommended endocervical sampling in non-pregnant women with no visible lesion or unsatisfactory colposcopy [25], implying all non-pregnant women who attended colposcopy have a histological sample recorded. The 2017 colposcopy guidelines recommend against non-targeted biopsies in the lowest risk women referred to colposcopy [26], but this was after the time of our study. Ten percent of women in the ‘screened – no colposcopy referral’ category may have been referred to colposcopy on the basis of a screening result indicating repeat testing at a short interval; when there were no prior test results to determine whether or not it was a first result indicating a repeat test at a short interval, we assumed it was their first result indicating repeat testing at a short interval if they did not have a biopsy within 6 months. It is possible that some women who were diagnosed with cervical cancer in New Mexico had screening tests that were not captured.

A third of women who weren't screened in a 3-year period had a positive screen in the 1–4 months prior to diagnosis; since we do not know why any screening or diagnostic test was carried out, we are not able to determine whether these women were attending routine screening or were tested due to symptoms. It is not possible to know whether specific cancers would have been prevented had the woman attended screening or colposcopy. Our estimate of the number of cancers that would be diagnosed in the absence of screening is likely an underestimate, since this estimate is based on not attending screening in a 3 year period, though some of these women will have attended screening with a longer interval, which will provide some protection.

Two-thirds of screening aged women who were diagnosed with cervical cancer in New Mexico had not attended screening in a 3-year period prior to diagnosis. This indicates that improving screening coverage has the most potential for reducing the incidence of cervical cancer among women above vaccination age. Failsafe failures were associated with almost one in every ten cancers, so there is a role for improved safety netting. 55% of cancers in screened women were diagnosed following a negative screen, indicating that increasing the sensitivity of the screening test would reduce cancer incidence. Very few cancers were a result of treatment failures.

## Funding

This work was supported by the 10.13039/100000054US National Cancer Institute (NCI) U54CA164336 to CMW (CMW, CLW, MR) with subcontracts to Texas A & M University, College Station, Texas (YJM, DWG) and to University of Alabama at Birmingham (ICS) and by the 10.13039/100000060US National Institute of Allergy and Infectious Diseases U19AI113187 to CMW with subcontract to Queen Mary University of London (QMUL) (JC, PDS). This project was also supported by Contract HHSN261201800014I, Task Order HHSN26100001 from the 10.13039/100000054National Cancer Institute (CLW). In addition, support was received from 10.13039/501100000289Cancer Research UK programme grants C8162/A16892 and C8162/A29083 to PDS (RL, CM) and C569/A16891 to JC, from NCI P30CA118100 (to CL Willman) (YJM) and the 10.13039/100000010Ford Foundation (YJM).

## Declaration of Competing Interest

JC and CMW have received funds from grants, cooperative agreements or subcontracts related to cervical screening and triage through their institutions. JC reports grants to his institution and personal fees from Qiagen, Becton Dickinson (BD), Genera Biosystems (GB), and grants to his institution from 10.13039/100015160Hologic, Gene First, and Trovagene, all outside the submitted work. CMW reports receiving reagents and equipment from Roche Molecular Systems, Roche Ventana Medical Systems and GB through her institution and personal fees from BD all outside of the submitted work. PDS reports collaborating in studies receiving reagents and equipment from Hologic and Roche Molecular Systems outside of the submitted work. RL, CM, CLW, MR, YJM, DWG, ICS have no interests to report. The findings and conclusions of this manuscript are those of the authors and do not necessarily represent the official position of the Centers for Disease Control and Prevention.

## Appendix A Supplementary data

Supplementary mateiral Image 1
