# Erratum to ‘A state-wide population-based evaluation of cervical cancers arising during opportunistic screening in the United States’ [Gynecologic Oncology 159 (2020) 344–353]

**DOI:** 10.1016/j.ygyno.2021.04.009

**Published:** 2021-06

**Authors:** Rebecca Landy, Christopher Mathews, Michael Robertson, Charles L. Wiggins, Yolanda J. McDonald, Daniel W. Goldberg, Isabel C. Scarinci, Jack Cuzick, Peter D. Sasieni, Cosette M. Wheeler, for the New Mexico HPV Pap Registry (NMHPVPR) Steering Committee Members

**Affiliations:** a Wolfson Institute of Preventive Medicine, Queen Mary University of London, London, United Kingdom; b Division of Cancer Epidemiology and Genetics, National Cancer Institute, National Institutes of Health, Department of Health and Human Services, Bethesda, MD, USA; c School of Cancer & Pharmaceutical Sciences, King's College London, London SE1 9RT, United Kingdom; d University of New Mexico Comprehensive Cancer Center, The Center for HPV Prevention, University of New Mexico Health Sciences Center, Albuquerque, NM, USA; e University of New Mexico Comprehensive Cancer Center, Department of Internal Medicine, University of New Mexico Health Sciences Center, Albuquerque, NM, USA; f Department of Human and Organizational Development, Vanderbilt University, Nashville, TN, USA; g Department of Geography, Texas A&M University, College Station, TX, USA; h Division of Preventive Medicine, University of Alabama at Birmingham, AL, USA; i Departments of Pathology and Obstetrics & Gynecology, University of New Mexico Health Sciences Center, Albuquerque, NM, USA

The publisher regrets that the New Mexico HPV Pap Registry (NMHPVPR) Steering Committee Members were not mentioned in the original version of the article. The online version has been updated and the correct author list can also be found above.

The publisher would like to apologise for any inconvenience caused.

