# Supplementary material for: A state-wide population-based evaluation of cervical cancers arising during opportunistic screening in the United States
Source: Gynecol Oncol. 2020 Nov;159(2):344–53. doi: 10.1016/j.ygyno.2020.08.033 (PMC7594931; doi:10.1016/j.ygyno.2020.08.033)
Supplement: Supplementary file 1 — Supplementary mateiral [file mmc1.docx]

**Supplementary Methods**

We estimated the proportion of cancers that could have been prevented in the absence of cervical screening or if all women had attended screening. We used results from previous analyses on the effectiveness of cervical screening on preventing cervical cancer in New Mexico; namely that the odds ratios for cervical cancer among women who attended screening in a 3-year period compared to women who did not were 0.62 (stage I) and 0.22 (stage II+). We used the observed numbers of stage I and stage II cancers, stratified by whether the woman was screened or unscreened, then weighted up to allow for missing stage information, giving a total number of stage I and stage II+ cancers. We assumed that the stage distribution was the same for women with and without stage information recorded, within screening classification (screened vs unscreened). The number of stage-specific cancers in the absence of screening was calculated by N_0_ + (N_1_/OR_1_), where N_0_ is the number of stage-specific cancers in unscreened women occurring with current screening coverage, N_1_ is the number of stage-specific cancers occurring in screened women under current screening coverage, and OR_1_ is the stage-specific OR of cervical cancer for women who attended screening relative to women who did not (0.62 for stage I, and 0.22 for stage II+). The number of stage-specific cancers assuming 100% screening coverage was calculated by N_1_ + (N_0_ * OR_1_).

To estimate the number of cancers that would have been associated with each screening history had there been 100% screening coverage, we first estimated the numbers of stage I and stage II+ cancers that would have been prevented and then the numbers that could be attributed to “failure” of different aspects of screening. We assume that the proportions of screened women who develop cancer in various categories is independent of the screening coverage. We further assumed that the stage distribution of women with unreported stage is the same as for women with stage reported, stratified by screening status.

**Supplementary Table 1**: Screening history classification for women diagnosed with cervical cancer aged 25-64 years in New Mexico, May 2009 - December 2016, dividing inadequately screened women by whether they had a negative test in the peri-diagnostic period, or no test in the peri-diagnostic period

|  | Inadequately screened  ***** | | Negative test in peri-diagnostic | | No test in peri-diagnostic | |
| --- | --- | --- | --- | --- | --- | --- |
|  | **N** | **Column %** | **N** | **Column %** | **N** | **Column %** |
| **Total** | 222 | 100% | 13 | 100% | 209 | 100% |
|  |  |  |  |  |  |  |
| **Age (years)** |  |  |  |  |  |  |
| **25-34** | 24 | 11% | 1 | 8% | 23 | 11% |
| **35-44** | 66 | 30% | 7 | 54% | 59 | 28% |
| **45-54** | 38 | 17% | 2 | 15% | 36 | 17% |
| **55-64** | 94 | 42% | 3 | 23% | 91 | 44% |
| **FIGO stage** |  |  |  |  |  |  |
| **IA** | 16 | 7% | 2 | 15% | 14 | 7% |
| **IB**** | 44 | 20% | 2 | 15% | 42 | 20% |
| **II+** | 136 | 61% | 7 | 54% | 129 | 62% |
| **Unknown** | 26 | 12% | 2 | 15% | 24 | 11% |
| **Morphology** |  |  |  |  |  |  |
| **Squamous** | 159 | 72% | 9 | 69% | 150 | 72% |
| **Adenocarcinoma** | 37 | 17% | 3 | 23% | 34 | 16% |
| **Other** | 26 | 12% | 1 | 8% | 25 | 12% |
| **Race/Ethnicity** |  |  |  |  |  |  |
| **Non-Hispanic White** | 90 | 41% | 3 | 23% | 87 | 42% |
| **White Hispanic** | 99 | 45% | 9 | 69% | 90 | 43% |
| **Native American** | 25 | 11% | 1 | 8% | 24 | 11% |
| **Other/unknown** | 8 | 4% | 0 | 0% | 8 | 4% |
| **Health insurance** |  |  |  |  |  |  |
| **Private** | 75 | 34% | 8 | 62% | 67 | 32% |
| **Medicaid** | 63 | 28% | 3 | 23% | 60 | 29% |
| **Medicare and other Government** | 18 | 8% | 0 | 0% | 18 | 9% |
| **Not insured** | 26 | 12% | 0 | 0% | 26 | 12% |
| **Unknown** | 40 | 18% | 2 | 15% | 38 | 18% |
| **RUCA***** |  |  |  |  |  |  |
| **Most urban (RUCA 1-3)** | 136 | 61% | 8 | 62% | 128 | 61% |
| **RUCA 4-7** | 63 | 28% | 3 | 23% | 60 | 29% |
| **Most rural (RUCA 8-10)** | 23 | 10% | 2 | 15% | 21 | 10% |

* For definitions of screening classifications, see Figures 1 and 2. ** FIGO stage IB includes stage I not otherwise specified. *** Rural-urban commuting area, based on address at diagnosis
